# A Case of Metastatic CNS Melanoma of Unknown Primary Presenting with Seizures

**DOI:** 10.1155/2022/3099750

**Published:** 2022-01-06

**Authors:** Viva Nguyen, Samar Aboulenain, Shawn Mohammed, Sahyli Perez Parra

**Affiliations:** ^1^Internal Medicine Resident, University of Miami JFK GME, Atlantis, FL, USA; ^2^Medical Specialists of the Palm Beaches Neurology, JFK Medical Center, Atlantis, FL, USA

## Abstract

Seizures are a common occurrence. The goal of evaluating a seizure is to identify the etiology and to determine the likelihood of recurrence as well as guide management. We present a unique presentation of a 47-year-old female that presented with late onset seizures admitted due to status epilepticus. Brain magnetic resonance indicated diffuse supratentorial hemorrhagic lesions. Neurological workup including brain vessel imaging, CT chest, abdomen, and pelvis as well as CSF and serological workup for vasculitis failed to demonstrate the cause of her brain lesions. Ultimately, a brain biopsy showed metastatic melanoma of unknown primary origin.

## 1. Introduction

Seizures are a common occurrence that affects 8 to 10 percent of the general population [1]. The primary goal in the management of seizures is to determine the etiology and evaluate the risk of having recurrent episodes. Seizures develop as a result of a sudden change in the electrical activity of neuronal networks in the cerebral cortex. Essentially, any acute or acute on chronic insult to the brain can lead to a seizure. Common causes include acute ischemic or hemorrhagic stroke, subdural hematoma, subarachnoid hemorrhage, traumatic brain injury, hypoxic-ischemic injury, brain abscess, brain tumor, meningitis or encephalitis, acute medical illness, metabolic disturbances, substance ingestion or withdrawal, and medication exposure [2].

## 2. Case Report

A 47-year-old White female with a past medical history of seizure disorder that started two years ago presented to the hospital with recurrent seizures. Two years ago, the patient presented with focal to generalized seizures while at work, witnessed by a bystander and recorded by the video system of the store. The patient was talking and suddenly developed slurred speech followed by head and truncal version to the right side and then fell to the ground with generalized tonic-clonic seizure. The patient denied any warning symptoms prior to her symptoms. She reported urine incontinence, tongue biting, and loss of consciousness with a clear postictal state. She denied any systemic symptoms including fever, weight loss, skin rash, or joint pain. The patient is a smoker with no known history of malignancy and has never completed any cancer screenings. She denied high-risk sexual behavior, sick contacts, or traveling outside the United States. The patient lived in Michigan for two years a while ago, and she denied any tick bites or history of Lyme disease. Physical exam was unremarkable for any focal neurological deficits, no palpable masses or lymph nodes, and no oral or obvious skin lesions. Magnetic resonance imaging (MRI) performed at that time showed increased signal in the left frontal lobe, negative for enhancement or restricted diffusion. MRA of the head at that time was without evidence of aneurysm, dissection, and intercranial atherosclerosis. Paraneoplastic antibodies were negative (including amphiphysin, ANNA-1, CRMP, PCA-1, and PCA-2), and lumbar puncture was negative (including HSV, CMV, VDRL, echovirus, coxsackie, VZV, adenovirus, West Nile virus, cryptococcal, toxoplasma, 14-3-3 protein tau). She was discharged on valproic acid to follow-up in the outpatient setting. Two months prior to the latest admission, she discontinued the valproic acid due to distorted vision and poor memory followed by an admission in an outside hospital after developing a seizure. Neuroimaging performed in the outside hospital showed more numerous intracranial lesions. A lumbar puncture performed at the time was unremarkable. Differential diagnoses at that time included meningoencephalitis with hemorrhage, infarcts with hemorrhagic transformation, metastatic disease, septic emboli, and paraneoplastic syndromes. She was again discharged on valproic acid and steroids and advised to follow-up for malignancy workup including obtaining a positive emission tomography (PET) scan. However, the patient was lost to follow-up due to lack of health insurance coverage.

**Figure 1:**
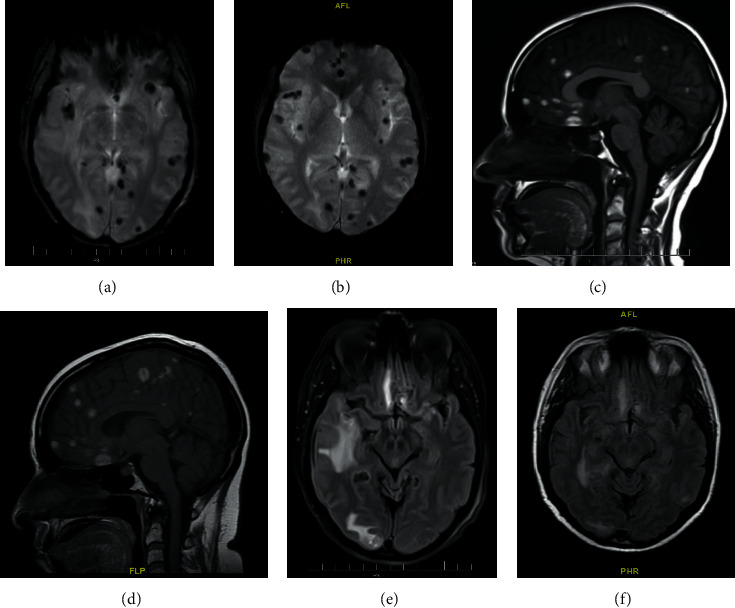
MRI brain. Innumerable foci of parenchymal hemorrhage throughout the bilateral cerebral hemispheres as well as left frontal subarachnoid hemorrhage, with mild edema and minimal local mass effect at these sites. Images (a), (c), and (e) are from a previous MRI brain (12/2020) and images (b), (d), and (f) are from the current MRI (02/2021).

On the day of this admission, the patient had three seizures at home witnessed by her partner, and then she had three more seizures in the emergency room despite receiving intravenous lorazepam. The patient was intubated and transferred to the intensive care unit for airway protection and management of status epilepticus. Over the course of her hospitalization, her seizures resolved, and she was extubated. Computed tomography (CT) scan of the brain revealed innumerable supratentorial foci of cortical hemorrhage. An MRI of the brain demonstrated diffuse lesions of the right and left cerebrum with surrounding vasogenic edema and minimal mass effect concerning for metastasis (Figure 1). As seen in Figure 1, the lesions appear to have gotten worse over a short period of time. CT of the chest showed chronic obstructive pulmonary disease and partially calcified nodule (1.2 × 1.3 cm) in the right upper lobe. CT of the abdomen and pelvis was negative for masses. CT angiogram of the head and neck showed multiple cortical and subcortical hemorrhages, but no evidence of intracranial aneurysm or vascular malformation.

**Figure 2:**
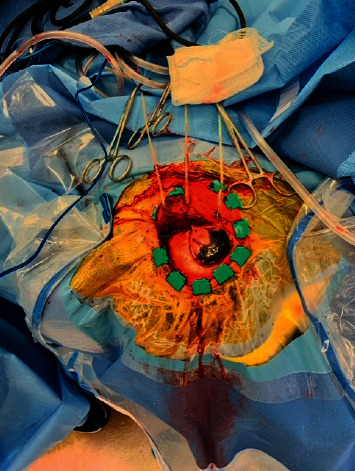
Brain biopsy procedure image.

The patient was transferred to our facility for conventional angiography to rule out primary central nervous system (CNS) vasculitis due to the unremarkable workup for malignancy. Her neurological and general exam were again completely unremarkable. Vasculitis serological markers were negative (Table 1). Four-vessel CT angiogram of the head did not show evidence of intracranial vascular abnormalities. Repeat lumbar puncture to rule out inflammatory changes in cerebral spinal fluid (CSF) showed elevated proteins, without pleocytosis or hypoglycorrhachia. The superior left temporal artery biopsy was negative for giant cell arteritis. The working diagnosis at that time was primary CNS vasculitis versus intracranial metastasis of unknown origin. A shared decision was made to undergo a cranial biopsy to aid the diagnosis. During the cranial biopsy procedure, a deeply pigmented lesion in a good plane from the gyrus concerning for melanoma was identified (Figure 2).

**Figure 3:**
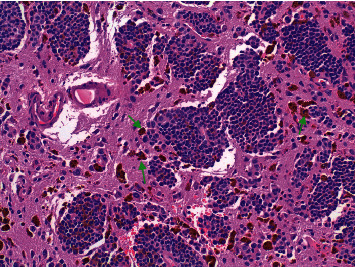
Brain biopsy. Glial tissue infiltrated by neoplastic melanocytic cells showing focally a perivascular pattern (see arrows). These cells show moderate pleomorphism, few mitoses, and focal melanin pigmentation.

**Figure 4:**
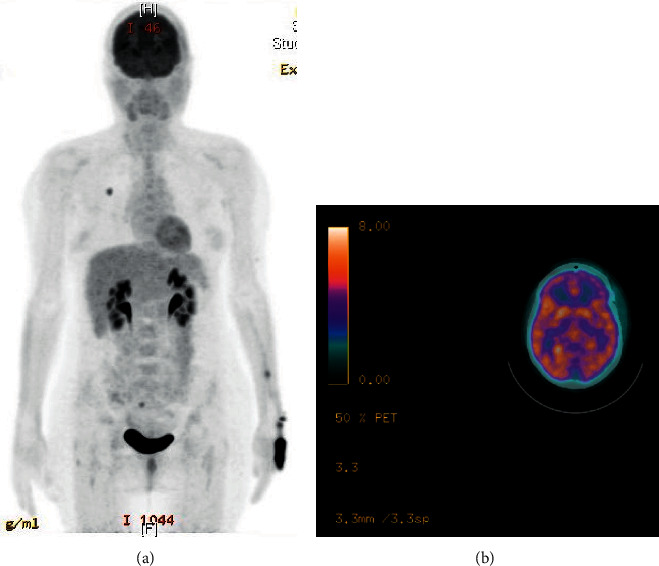
PET scan. Postsurgical changes are present in the left frontal parietal lobe. Punctate hypermetabolic foci are present in both cerebral hemispheres and cerebellum compatible metastatic lesions. There is a 1.2 cm hypermetabolic nodule in the right upper lobe with a max SUV of 4.3.

Brain biopsy of the largest lesion revealed a metastatic malignant melanoma with immunostains positive for S-100, HMB-45, and MART-1 (Figure 3). The specimen was also positive for BRAF mutation by immunohistochemistry. Lactate hydrogenase (LDH) was within normal limits, indicating a favorable prognosis. PET scan was performed revealing a 1.2 cm hypermetabolic nodule in the right upper lobe suspicious for lung cancer in addition to metastatic brain lesions (Figure 4). After the diagnosis of metastatic melanoma was confirmed in our patient, whole brain radiation was deemed of the utmost importance in order to conserve the CNS. BRAF mutation was positive, offering therapeutic potential for immunotherapy and chemotherapy. With the innumerable brain lesions, the patient was not a surgical candidate and thus would benefit best from radiotherapy and chemotherapy.

Ultimately, after health insurance was arranged, the patient was discharged home to begin whole brain radiation and initiate chemotherapy for metastatic melanoma of unknown origin. Since the most life-threatening disease process was the brain lesions, the decision was made to treat the brain lesions first and monitor the response of the lung nodule in the outpatient setting.

## 3. Discussion

The etiology of seizures is innumerous, including CNS vasculitides and brain tumors. Vasculitis is inflammation of any blood vessel, meaning it can affect any location in the body. The inflammation compromises the integrity of blood vessels, leading to potential swelling and scarring. Vessels can be narrowed, occluded, or ruptured, causing bleeding into surrounding tissues. Nervous system complications from vasculitis can include persistent headaches, thrombosis, strokes, confusion, abnormal sensations, muscle weakness, seizures, vision, and speech difficulties [3]. Seizures can be secondary to epilepsy, which can be primary or secondary. Secondary causes of epilepsy are any lesions within the CNS (hemorrhage, ischemia, demyelination, tumor, or mass), infections, metabolic derangements, etc.

Our patient had a known history of seizures of two years, with no clear etiology previously. Her situation was complicated by the lack of health insurance. Her seizures were initially controlled while on anticonvulsant medication. Physical exam was unremarkable for any focal neurological deficits, no palpable masses or lymph nodes, and no oral or obvious skin lesions. The neuroimaging studies upon admission demonstrated multiple lesions concerning for hemorrhages or metastatic disease. The CT chest revealed a single upper right lobe nodule (1.2 × 1.3 cm), while the CT abdomen and pelvis was negative for any malignancy. The negative workup for vasculitis suggested an alternative diagnosis. Ultimately brain biopsy demonstrated a deeply pigmented lesion suggestive of a melanoma, which was confirmed by histopathological evaluation. Later, the PET scan revealed the metastatic brain lesions along with the lung nodule.

Primary CNS vasculitis along with metastatic brain tumor was initially at the top of the differential diagnoses. Other pathologies were considered in the differential diagnosis. Intravascular lymphoma could have a similar presentation but deemed unlikely given the lack of progression since symptoms onset two years ago [4]. Familial cerebral amyloid angiopathy can present in middle to late middle age with intracranial hemorrhage which is usually lobar and is associated with dementia and other neurological deficits in addition to family history [5]. Other mimickers of vasculitis including infectious causes (endocarditis, hepatitis B and C, and HIV), thromboembolic disease (antiphospholipid syndrome), hypercoagulable states (TTP), vasospastic disorders (drug exposures), multisystemic inflammatory disorders (sarcoidosis), or other malignancies (leukemia) [6] were included in the differential diagnoses.

Melanoma accounts for approximately 10 percent of all brain metastases, commonly associated with supratentorial disease [7]. Common symptoms include headache, neurological deficits, and/or seizures, such as in our patient. Malignant melanoma is typically diagnosed at an early stage, when excision can be curative. However, some patients present with metastatic disease as in our patient. The brain metastases are at a high risk of spontaneous hemorrhages. Metastatic melanoma of unknown primary occurs rarely, approximately 3.2% of all melanomas [8]. Melanoma of unknown primary (MUP) occurs more often in males in the fourth and fifth decades of life, which highlights the rarity of this case presentation [8]. Men have an increased likelihood for primary site regression due to reasons such as ignoring a primary cutaneous melanoma until complete regression and later presents as metastatic disease [9]. The majority of MUP is diagnosed in lymph nodes, followed by subcutaneous sites, and least commonly diagnosed in visceral organs [9]. MUP can also present as a paraneoplastic syndrome, reported as retinopathy, systemic vasculitis, and inflammatory demyelinating polyneuropathy [9, 10]. Similar to melanoma of known primary, the patient's age, AJCC stage, lactate dehydrogenase level, and number of metastases at diagnosis are all independent prognostic factors for MUP [11]. Patients with metastatic melanoma should undergo evaluation of the extent of the disease due to the possible presence of unsuspected lesions with whole body imaging and serum lactate dehydrogenase, which has important prognostic implications.

Stage IV MUP warrants aggressive therapy, and it should be treated similar to stage IV melanoma with known primary with a combination of surgery, chemotherapy, immunotherapy, and radiotherapy [12]. All patients with advanced melanoma should be evaluated for the presence of absence of a driver mutation at the V600 site *BRAF* gene, which promotes oncogenesis. For patients with disseminated metastases, immunotherapy such as checkpoint inhibition with antiprogrammed cell death 1 (anti-PD-1) antibodies alone or in combination with ipilimumab and targeted therapy with inhibiting the mitogen-activated protein kinase [MAPK] pathway have proven clinical roles in the treatment process. Advances in CNS therapy such as neurosurgical techniques, radiation therapy, and stereotactic radiosurgery have led to improvement in ability to control brain metastases, and systemic therapy with immunotherapy and BRAF plus MEK inhibitory therapy has significantly prolonged overall survival [13].

For our patient, the seizures were secondary to the brain lesions. Metastasis can present with focal neurological symptoms or seizures, demonstrated by our patient. Since the utmost importance was the metastatic brain lesions, the objective was to treat the brain lesions to prevent life threatening seizures, while monitoring the response of treatment to the lung nodule in the outpatient setting.

## 4. Conclusion

This case report demonstrates the importance of pursuing a complete evaluation in patients with new onset seizures as well as how diverse the etiology of seizures can be. When there is a medical mystery, consider metastatic melanoma as a potential diagnosis for an etiology of seizures. This case also highlights the impact that socioeconomic status can have on healthcare, as the diagnosis was delayed due to health insurance issues.

## Figures and Tables

**Table 1:** Vasculitis workup.

Test	Result
HIV antigen/antibody	Nonreactive
ESR	15 (normal 0–20 mm/hr)
CRP	1.3 (normal 0–1.0 mg/dL)
Lyme disease antibody (CSF)	Negative
Varicella zoster antibody (CSF)	Negative
Herpes virus 1 and 2 DNA PCR	Negative
Toxoplasmosis IgG CSF	Negative
VDRL (CSF)	Nonreactive
Rheumatoid factor	Negative
ANA	Negative
Anti-SSA/SSB	Negative
RNP antibody	Negative
SM antibody	Negative
Syphilis	Negative
C-ANCA	Negative
P-ANCA	Negative
Toxoplasma IgM and IgG	Negative

## Data Availability

The data used to support the findings of this study are available from the corresponding author upon request.
